# VPS13B maintains lysosomal homeostasis through regulation of TFEB

**DOI:** 10.1186/s13041-026-01309-y

**Published:** 2026-05-08

**Authors:** Soo-Kyeong Lee, Semin Park, Min-Young Yeom, Jin-A Lee

**Affiliations:** Department of Biological Sciences and Biotechnology, College of Life Sciences and Nanotechnology, Hannam University, 1646 Yuseong-daero, Yuseong-gu, Daejeon, 34054 Korea

**Keywords:** VPS13B, Cohen syndrome, Lysosome, TFEB, Lysosomal acidification, Neurodevelopmental disorder

## Abstract

Cohen syndrome (CS) is a rare autosomal recessive neurodevelopmental disorder characterized by intellectual disability, microcephaly, retinal dystrophy, and neutropenia. We previously demonstrated that VPS13B mediates phosphatidylinositol 4-phosphate (PI4P) transport to promote mitochondrial fission. Here, we identify VPS13B as a regulator of lysosomal homeostasis. VPS13B knockout (KO) HeLa cells exhibited aberrant lysosomal distribution and reduction in LAMP1-positive lysosomes. Bulk RNA sequencing revealed coordinated downregulation of lysosome-related genes, including genes required for acidification and lysosome biogenesis, which was confirmed by quantitative RT-PCR. Consistent with these transcriptional changes, VPS13B KO significantly reduced the abundance of LysoTracker-positive acidic compartments. Induced neurons derived from CS patient iPSCs recapitulated the loss of acidic lysosomal compartments, supporting disease relevance. Mechanistically, VPS13B KO altered TFEB mRNA levels and modestly increased the basal nuclear-to-cytoplasmic (N/C) ratio of endogenous TFEB, but blunted its further increase upon Torin1 treatment. Together, these findings identify VPS13B as a regulator of lysosomal homeostasis and provide insight into how VPS13B deficiency may contribute to Cohen syndrome pathology.

**Main text**

Cohen syndrome (CS) is a rare autosomal recessive disorder caused by mutations in the VPS13B gene, presenting with characteristic features including intellectual disability, microcephaly, retinal dystrophy, and neutropeni [1–3] a. VPS13B encodes a large protein belonging to the VPS13 family, which functions in lipid transport at membrane contact sites between organelles [4]. While our previous work established VPS13B’s role in PI4P transport and mitochondrial fission [5], its involvement in other organelles including lysosomes, functions remains incompletely understood.

Lysosomes are critical membrane-bound organelles responsible for cellular degradation, nutrient sensing, and metabolic regulation [6]. Lysosomal dysfunction has been implicated in numerous neurodegenerative and neurodevelopmental disorders [7]. Transcription factor EB (TFEB) serves as the master regulator of lysosomal biogenesis and autophagy, with its activity tightly controlled by mTORC1-mediated phosphorylation [8]. Upon mTORC1 inhibition, TFEB translocates to the nucleus and activates the coordinated lysosomal expression and regulation (CLEAR) network, promoting expression of lysosomal genes [9].

Given the multifaceted clinical manifestations of Cohen syndrome and the emerging importance of lysosomal function in neurodevelopmental disorders, we investigated whether VPS13B deficiency affects lysosomal homeostasis. Here, we report that VPS13B is essential for maintaining proper lysosomal distribution, abundance, and TFEB-mediated transcriptional regulation of lysosomal genes.

Consistent with previous studies, we observed abnormal organelle positioning in VPS13B knockout (KO) HeLa cells. While prior research characterized the hyperfused morphology of mitochondria, the loss of VPS13B which localizes to the Golgi was anticipated to alter the morphology of endosomes, late endosomes, and the Golgi(5). However, we unexpectedly observed perinuclear clustering of lysosomes, accompanied by a significant reduction in the number of LAMP1-positive lysosomes in VPS13B KO cells (Fig. 1A, B).

**Fig. 1:**
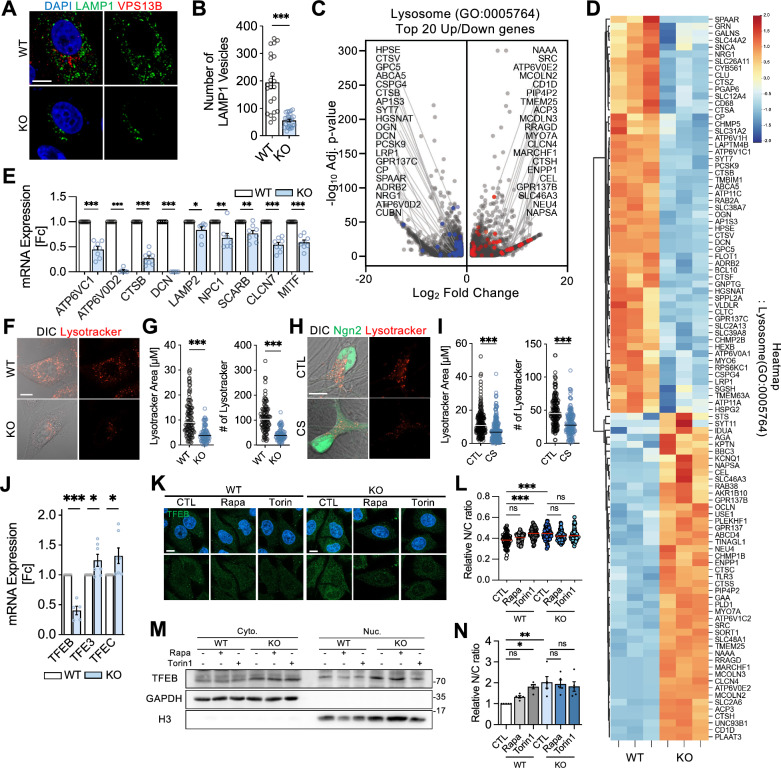
Lysosomal dysfunction through altered TFEB regulation in VPS13B depleted cell **A** Confocal images of Wild-type(WT) and VPS13B Knockout(KO) cells expressing EGFP-LAMP1 and stained with VPS13B antibodies and DAPI. Scale bar = 10 µm. **B** Quantification of LAMP1 vesicles number in WT and VPS13B KO cells. Analysis via unpaired t-test. ***p < 0.001. WT; n = 22, KO; n = 26. All data are obtained from 3 independent experiments. **C** Volcano plot displays gene expression changes between WT and VPS13B KO groups. Red and Blue points indicate upregulated and downregulated genes associated with the lysosome (GO:0005764), respectively. Grey points represent non-lysosome genes. Black points denote the top 20 genes with the most significant changes, which are labeled. **D** Heatmap shows the relative expression of lysosome-associated genes (GO:0005764; n = 104) that differ significantly between WT and VPS13B KO groups (P-adj < 0.05). Colors represent row-scaled Z-scores, with red indicating higher expression and blue indicating lower expression. **E** Quantification of mRNA expression in WT and VPS13B KO cells. Analysis via Unpaired t-test. *p > 0.01, **p > 0.001, ***p < 0.001. All data are obtained n > 3 independent experiments. **F** Confocal images of WT and VPS13B KO cells stained with Lysotracker. Scale bar = 10 µm. **G** Quantification of Lysotracker area and number in WT and VPS13B KO cells. Analysis via Unpaired t-test. ***p < 0.001. WT; n = 99, KO; n = 86. All data are obtained from 3 independent experiments. **H** Control (CTL) and Cohen syndrome patient (CS) induced Neuron(iN) cells induced via EGFP-Ngn2 and rtTA, stained with Lysotracker. Scale bar = 10 µm. **I** Quantification of Lysotracker area and number in Control and CS iN cells. Analysis via Unpaired t-test. ***p < 0.001. Area; CTL; n = 171, CS; n = 174, number; CTL; n = 201, CS; n = 235. All data are obtained from 5 independent experiments. **J** Quantification of TFEB mRNA expression in WT and VPS13B KO cells. Analysis via unpaired t-test. ***p < 0.001. Data are obtained n = 5 independent experiments. **K** Confocal images of endogenous TFEB in WT and VPS13B KO cells. Scale bar = 10 µm. **L** Quantification of TFEB translocation ratio(Nucleus intensity / Cytosol intensity) in WT and VPS13B KO cells. Analysis via One-way ANOVA. ***p < 0.001. WT CTL; n = 63, WT Rapa; n = 59, WT Torin1; n = 71, KO CTL; n = 71, KO Rapa; n = 58, KO Torin1; n = 66. All data are obtained 3 independent experiments. **M** Nucleus fraction of TFEB in WT and VPS13B KO cells. **N** Quantification of TFEB nucleus translocation. Analysis via One-way ANOVA. *p > 0.01, **p > 0.001. Data are obtained 5 independent experiments

Next, we performed RNA sequencing (RNA-seq) on Wild-Type (WT) and VPS13B KO cells. Gene Ontology (GO) analysis revealed that genes associated with the lysosome (GO:0005764) were significantly differentially expressed in the VPS13B KO cells. Volcano plot and Heatmap analysis of the top 20 down-regulated/up-regulated genes clearly demonstrated reduced expression levels in VPS13B KO cells (Fig. 1C, D). Quantitative RT-PCR validation confirmed significant downregulation of nine key lysosomal genes, including ATP6V1C1, ATP6V0D2 (V-ATPase subunit), CTSB (cathepsin B), DCN (lysosomal activity regulator), LAMP2 (lysosomal membrane protein), NPC1 (cholesterol transporter), SCARB2 (scavenger receptor), CLCN7 (chloride channel), and MITF (transcription factor) (Fig. 1E). This transcriptomic signature suggests that VPS13B deficiency impairs the transcriptional program governing lysosomal biogenesis and function. The downregulation of ATP6V1C1 and ATP6V0D2, which encodes subunit of the vacuolar H⁺-ATPase responsible for lysosomal acidification, is particularly noteworthy as it may directly contribute to the functional defects observed below. Similarly, reduced expression of LAMP2, a major lysosomal membrane protein, may explain the decreased number of LAMP1-positive lysosomes. The downregulation of MITF, a transcription factor that regulates lysosomal gene expression alongside TFEB, suggests broader disruption of the lysosomal transcriptional network.

Given the transcriptomic changes, we next assessed lysosomal status in VPS13B KO cells using LysoTracker, a fluorescent probe that accumulates in acidic compartments. VPS13B KO cells exhibited a significant reduction in both the number and area of LysoTracker-positive compartments compared to control cells under basal conditions, indicating a decreased abundance of acidic lysosomal compartments (Fig. 1F, G). This reduction in acidic compartments it is likely that both mechanisms contribute, though direct measurement of lysosomal pH will be required to confirm the acidification, reduced lysosome number or both. Given the observed reduction in LAMP1-positive lysosomes (Fig. 1B) and downregulation of ATP6V1C1 and ATP6V0D2 (Fig. 1E), it is likely that both mechanisms contribute to the decreased LysoTracker signal.

This defect was not limited to KO cell lines; induced neurons (iNs) derived from Cohen syndrome patient-derived iPSCs also exhibited a significant reduction in LysoTracker-positive lysosomal compartments (Fig. 1H, I), demonstrating the clinical relevance of this finding. This reduction in acidic lysosomal compartments may compromise degradative capacity and contribute to the cellular pathologies associated with Cohen syndrome, potentially underlying some of the neurological manifestations of the disease.

Quantitative RT-PCR analysis of MiT/TFE family members showed altered TFEB mRNA expression in VPS13B KO cells, while TFE3 and TFEC mRNA levels were modestly increased (Fig. 1J). Consistently, lysosome-associated genes, including LAMP2, CTSB, and MITF, remained significantly downregulated (Fig. 1E). These data suggest that VPS13B KO does not broadly suppress the MiT/TFE family. However, this potential compensatory response was insufficient to restore lysosomal transcriptional output.

Our findings establish a previously unrecognized link between VPS13B and lysosomal function, thereby expanding the mechanistic framework underlying Cohen syndrome pathogenesis. In line with growing evidence that lysosomal dysfunction is a shared feature of neurodevelopmental disorders [10], VPS13B KO cells and patient-derived neurons exhibited a reduced number of lysosomes, diminished acidic compartments, prompting us to examine whether TFEB activity was altered in VPS13B KO cells.

Endogenous TFEB imaging showed a modestly increased basal nuclear-to-cytoplasmic (N/C) ratio in VPS13B KO cells (Fig. 1K, L), but this was not accompanied by increased lysosomal gene expression. Rapamycin did not significantly alter the TFEB N/C ratio in WT cells, whereas Torin1 increased this ratio in WT cells but not further in VPS13B KO cells. Consistent with these imaging data, subcellular fractionation followed by western blot analysis confirmed that basal nuclear TFEB levels were elevated in VPS13B KO cells compared to WT cells, and that Torin1-induced nuclear accumulation of endogenous TFEB was attenuated in VPS13B KO cells compared to WT cells, while Rapamycin showed no significant effect in either condition (Fig. 1M, N). Together, these data suggest that VPS13B KO alters basal TFEB distribution and reduces lysosomal transcriptional output.

In neurons, such defects may compromise lysosomal and proteostatic capacity during development, thereby contributing to Cohen syndrome-associated neurological phenotypes. The perinuclear clustering and reduced number of lysosomes in VPS13B KO cells may reflect disrupted organellar trafficking, tethering, or biogenesis. Given VPS13B’s role in lipid transport at membrane contact sites [5, 11], VPS13B KO may impair the lipid composition or membrane dynamics required for proper lysosomal positioning and formation. The downregulation of multiple lysosomal genes, including the transcription factor MITF, suggests a coordinated transcriptional defect that may involve dysregulation of the broader MiT/TFE transcriptional network, including TFEB.

This study demonstrates that VPS13B is essential for maintaining lysosomal distribution, abundance, and TFEB-mediated transcriptional regulation. VPS13B KO leads to perinuclear clustering of lysosomes, a marked reduction in lysosome number, downregulation of key lysosomal genes, and decreased abundance of acidic lysosomal compartments in both cultured cells and patient-derived neurons. These lysosomal defects are accompanied by altered TFEB mRNA expression, a modest increase in the basal nuclear-to-cytoplasmic ratio of endogenous TFEB, and impaired further increase in this ratio upon Torin1 treatment. Importantly, the modest increases in TFE3 and TFEC expression suggest that compensatory changes in other MiT/TFE family members are insufficient to rescue lysosomal gene expression in VPS13B KO cells. Although the precise molecular link between VPS13B and TFEB regulation awaits further clarification, our findings uncover a lysosome-centered cellular vulnerability in Cohen syndrome and identify lysosomal dysfunction as a potential therapeutic entry point for this neurodevelopmental disorder.

## Data Availability

All data supporting the findings of this study are available from the corresponding authors upon reasonable request.

## Abbreviations

CS: Cohen syndrome
GO: Gene ontology
iNs: Induced neurons
iPSCs: Induced pluripotent stem cells
KO: Knockout
CLEAR network: Coordinated lysosomal expression and regulation network
LAMP1: Lysosomal-associated membrane protein 1
mTORC1: Mechanistic target of rapamycin complex 1
PI4P: Phosphatidylinositol 4-phosphate
Rapa: Rapamycin
RNA-seq: RNA sequencing
TFEB: Transcription factor EB
VPS13B: Vacuolar protein sorting 13 homolog B

## References

[1] Cohen MM, Hall BD, Smith DW, Graham CB, Lampert KJ. A new syndrome with hypotonia, obesity, mental deficiency, and facial, oral, ocular, and limb anomalies. J Pediatr. 1973;83:280–4. 4717588 10.1016/s0022-3476(73)80493-7

[2] Rodrigues JM, Fernandes HD, Caruthers C, Braddock SR, Knutsen AP. Cohen syndrome: review of the literature. Cureus. 2018;10:e3330. 30473963 10.7759/cureus.3330 PMC6248805

[3] Kolehmainen J, et al. Cohen Syndrome is caused by mutations in a novel gene, COH_1_, encoding a transmembrane protein with a presumed role in vesicle-mediated sorting and intracellular protein transport. Am J Hum Genet. 2003. 10.1086/375454. 12730828 10.1086/375454 PMC1180298

[4] Kumar N, et al. VPS13A and VPS13C are lipid transport proteins differentially localized at ER contact sites. J Cell Biol. 2018;217:3625–39. 30093493 10.1083/jcb.201807019 PMC6168267

[5] Lee S-K, et al. VPS13B recruits lipid vesicles to promote mitochondrial fission and quality control. Nat Commun. 2025. 10.1038/s41467-025-67445-6. 41402289 10.1038/s41467-025-67445-6 PMC12820316

[6] Ballabio A, Bonifacino JS. Lysosomes as dynamic regulators of cell and organismal homeostasis. Nat Rev Mol Cell Biol. 2020;21:101–18. 31768005 10.1038/s41580-019-0185-4

[7] Platt FM, D’azzo A, Davidson BL, Neufeld EF, Tifft CJ. Lysosomal storage diseases. Nat Rev Dis Primers. 2018;4:27. 30275469 10.1038/s41572-018-0025-4

[8] Settembre C, et al. A lysosome-to-nucleus signalling mechanism senses and regulates the lysosome via mTOR and TFEB. EMBO J. 2012;31:1095–108. 22343943 10.1038/emboj.2012.32 PMC3298007

[9] Roczniak-Ferguson A, et al. The transcription factor TFEB links mTORC1 signaling to transcriptional control of lysosome homeostasis. Sci Signal. 2012;5:ra42–ra42 10.1126/scisignal.2002790 PMC3437338 22692423

[10] Carpentieri G, et al. Dominantly acting variants in ATP6V1C1 and ATP6V1B2 cause a multisystem phenotypic spectrum by altering lysosomal and/or autophagosome function. HumanGene Genomics Adv. 2024;5:100349. 10.1016/j.xhgg.2024.100349 10.1016/j.xhgg.2024.100349 PMC11465052 39210597

[11] Fraldi A, et al. Lysosomal fusion and SNARE function are impaired by cholesterol accumulation in lysosomal storage disorders. EMBO J. 2010;29:3607–20. 20871593 10.1038/emboj.2010.237 PMC2982760

